# Protoplast-mediated transformation of *Madurella mycetomatis* using hygromycin resistance as a selection marker

**DOI:** 10.1371/journal.pntd.0012092

**Published:** 2024-04-05

**Authors:** Saskia du Pré, Mickey Konings, Dorenda J. A. Schoorl, Ahmed H. Fahal, Mark Arentshorst, Arthur F. J. Ram, Wendy W. J. van de Sande

**Affiliations:** 1 Erasmus MC, Department of Medical Microbiology and Infectious Diseases, Rotterdam, The Netherlands; 2 Mycetoma Research Centre, Khartoum, Sudan; 3 Molecular Microbiology and Biotechnology, Institute of Biology Leiden, Leiden University, Leiden, The Netherlands; Cornell University, UNITED STATES

## Abstract

*Madurella mycetomatis* is the main cause of mycetoma, a chronic granulomatous infection for which currently no adequate therapy is available. To improve therapy, more knowledge on a molecular level is required to understand how *M*. *mycetomatis* is able to cause this disease. However, the genetic toolbox for *M*. *mycetomatis* is limited. To date, no method is available to genetically modify *M*. *mycetomatis*. In this paper, a protoplast-mediated transformation protocol was successfully developed for this fungal species, using hygromycin as a selection marker. Furthermore, using this method, a cytoplasmic-GFP-expressing *M*. *mycetomatis* strain was created. The reported methodology will be invaluable to explore the pathogenicity of *M*. *mycetomatis* and to develop reporter strains which can be useful in drug discovery as well as in genetic studies.

## Author summary

*Madurella mycetomatis* is the main cause of mycetoma, a Neglected Tropical Disease characterized by large tumorous swellings. Currently little is known about the role of the pathogen in mycetoma formation. Furthermore, there are only a few drugs which are able to inhibit this fungus. In order to gain insight in the disease mycetoma and identify novel drug targets we need a method to genetically modify *M*. *mycetomatis*. Up till now this was not available for *M*. *mycetomatis*. In this paper, we developed a method in which we for the first time were able to genetically modify *Madurella mycetomatis*. We did this by removing the *M*. *mycetomatis* cell wall and introduced a plasmid into the fungal cell which made the fungus express GFP. The reported methodology can be adapted to introduce other reporters in *M*. *mycetomatis* and will be invaluable to explore the pathogenicity of *M*. *mycetomatis*.

**Key Points:**

- A genetic transformation method for *Madurella mycetomatis* has been developed.
- Hygromycin is a suitable selection marker for *Madurella mycetomatis*.
- GFP can be used as a reporter for *Madurella mycetomatis*.

## Introduction

*Madurella mycetomatis* is the main causative agent of mycetoma, a chronic and granulomatous infection of the subcutaneous tissue [1,2]. The disease is highly endemic in tropical and subtropical regions and is characterized by large subcutaneous swellings in which grains are present. Grains consist of a hard cement-like layer in which the pathogen becomes embedded. The current treatment involves a combination of antifungal therapy and surgery, but the success rates are low. To improve therapy, more knowledge is required on the molecular processes in *M*. *mycetomatis* leading towards grain formation. By using proteomic and transcriptomic studies to unravel the processes involved in grain formation, candidate genes potentially involved in the pathogenesis of *M*. *mycetomatis* have been identified [3,4]. However, no molecular toolbox for *M*. *mycetomatis* was available to characterize these genes. Therefore, the aim of this study was to develop a method to genetically modify *M*. *mycetomatis*. With this tool, knock-out strains can be created for genes of interest to study their role in virulence, and proteins can be tagged with GFP to study their localization within *M*. *mycetomatis* hyphae. In addition, GFP could be used to quantify minimal inhibitory concentration assays (MICs) and visualize infections in animal models or cell cultures.

Genetic engineering of various fungal species, including for *Chaetomium* species, a close relative of *Madurella*, is routinely performed and has helped in understanding the function of genes, cellular processes, and virulence factors [5]. One of the most commonly applied techniques for the genetic manipulation of filamentous fungi is protoplast-mediated transformation [6,7]. Protoplasts are fungal cells from which the fungal cell wall has been enzymatically removed. The cell wall represents a physical barrier for the transforming DNA to enter the fungal cell, therefore, removing it facilitates polyethylene glycol (PEG)-mediated uptake of this DNA. This method is relatively easy to apply and does not require expensive materials, which is why the technique can be applied in most laboratories. Furthermore, this method is also suitable for a non-sporulating fungal species, such as *M*. *mycetomatis*. In this paper, a protoplast-mediated transformation method is described that has been optimized for *M*. *mycetomatis*. The availability of this method opens up new opportunities to unravel the molecular mechanisms behind *M*. *mycetomatis*-induced mycetoma infections, which will help to identify novel drug targets for treating mycetoma.

## Methods

### Strains and culture conditions

*M*. *mycetomatis* strain Mm55 was used to generate the genetically manipulated strains described in this manuscript. Strain Mm55 was isolated in Sudan in 1999 [8]. Mm55 was maintained on Sabouraud Dextrose (SAB) Agar (Difco). For a liquid culture, 1/3 of a 2–4 week old colony was harvested, placed in 10 mL Mueller-Hinton II (MH) broth (Difco) and sonicated for 10 s at 10 μm (Soniprep 150 plus, MSE, UK). The culture was incubated at 37°C for 5–7 days, after which it was sonicated again and used to prepare a fresh culture in 50 mL MH with a transmission of 70% at 660 nm (Novaspec II, Parmacia Biotech). The 70% transmission culture was incubated for 7 days at 37°C after which the mycelium was harvested for use in subsequent experiments (transformation or RNA isolation).

For growth assays on agar plates, circles were cut with the back of pipet tip from 2–4 week old colonies of the required strains and placed in the middle of a MH or SAB plate, which were then incubated at 37°C. To investigate whether hygromycin B could be used as a selection marker for *M*. *mycetomatis*, a growth test was conducted to investigate its susceptibility against hygromycin B. Hygromycin B (Sigma) was dissolved in water to a stock concentration of 50 mg/ml. MH agar plates were prepared with different concentrations of hygromcyin B: 0, 10, 20 and 40 μg/ml. Mm55 was inoculated on the MH plates containing hygromycin, which were then incubated for two weeks at 37°C. Growth or lack of growth was observed visually.

### qPCR

RNA was isolated from one week old *M*. *mycetomatis* cultures. Mycelium was suspended in TRIzol Reagent (Thermo Fisher) and, using metal beads, was lysed with a tissue lyzer (Qiagen). RNA was extracted using chloroform, and precipitated and washed with ethanol. The RNA pellet was air-dried and suspended in diethyl pyrocarbonate (DEPC)-treated water. Remaining DNA was removed with the Ambion DNA-free kit. cDNA was prepared using the High Capacity cDNA Reverse Transcription Kit (Applied Biosystems). Expression of eight genes, suspected to be highly expressed in *M*. *mycetomatis*, was measured via real-time qPCR on a LightCycler 480 (Roche, Woerden, Netherlands). Genes and corresponding primers are listed in S1 Table. Each 20 μl qPCR reaction contained 10 μl of Lightcycler 480 mastermix (Roche), 0.2 μl of each primer, 1 μl SYTO82 (Roche) and 1 μl cDNA.

### DNA and cloning procedures

To isolate plasmid DNA the GENEjet miniprep kit (Thermo Scientific) was used. Genomic DNA of the fungal strains was isolated using the Quick-DNA Fungal/Bacterial miniprep kit (Zymo). DNA concentrations were measured with NanoDrop One (Thermo Scientific). PCR reactions were conducted with either Phusion polymerase (Thermo Scientific) or Q5 polymerase (NEB). See S2 Table for primer sequences. FastDigest restriction enzymes (Thermo Scientific) were used for digestions. PCR products and digestions were cleaned with the DNA clean & concentrator kit-5 (Zymo research). For ligation reactions, T4 ligase (Invitrogen) was used in overnight reactions at 16°C. Ligations were transformed into TOP10 *Escherichia coli* cells (Invitrogen). *E*. *coli* strains were maintained on LB medium supplemented with 50 μg/ml ampicillin (Sigma).

### Plasmids

Please refer to S3 Table for the plasmids used in this study. To generate the *M*. *mycetomatis*-optimized hygromycin resistance cassette, the *Aspergillus nidulans gpdA* promoter and *trpC* terminator in plasmid pAN7-1 [9] were replaced with the *M*. *mycetomatis gpdA* promoter and *trpC* terminator. The *A*. *nidulans gpdA* promoter was released from pAN7-1 with a BglII/SalI double digestion (1806 bp). The *A*. *nidulans trpC* terminator was released from pAN7-1 with a BamHI/*Hin*dIII double digestion (783 bp). The *M*. *mycetomatis gpdA* promoter was amplified with primer pair PgpdA-BglII_FW/PgpdA-SalI_RV (1502 bp) and the *trpC* terminator was amplified with primer pair TtrpC-BamHI_FW/TtrpC-HindIII_RV (497 bp), with the appropriate restriction sites attached to the primers. The PCR products were digested and then ligated into the digested pAN7-1 vector. The resulting plasmid was named pSDP002 (F 1).

**Fig 1:**
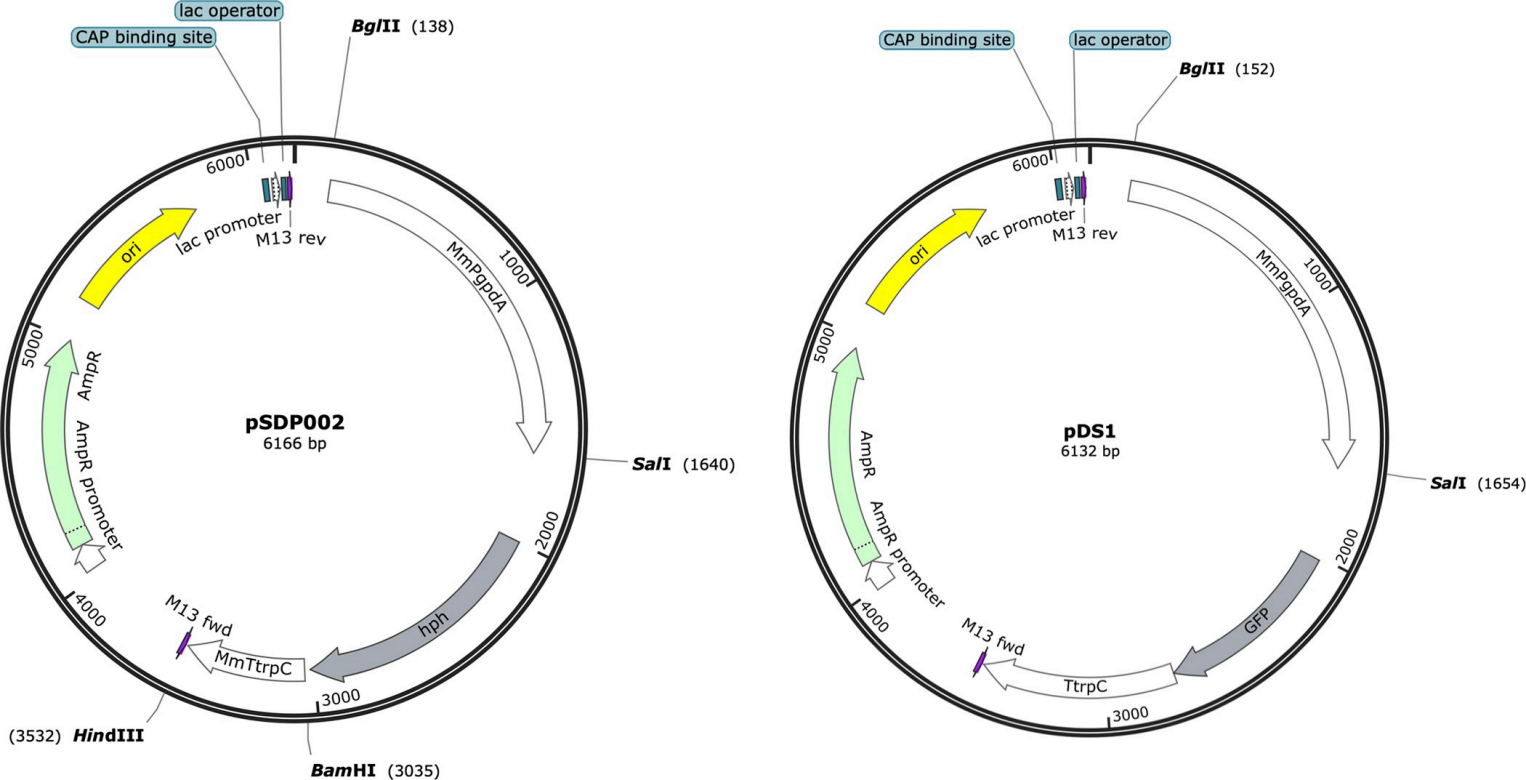
Plasmid maps for pDS1 and pSDP002.

To generate the *M*. *mycetomatis*-optimized GFP labelling cassette, the *Aspergillus gpdA* promoter in plasmid pGPD-eGFP was replaced with the *M*. *mycetomatis gpdA* promoter as described above for pAN7-1. The resulting plasmid was named pDS1 (Fig 1).

### Protoplast-mediated transformation

The mycelium was harvested and washed with sterile dH_2_O and added to a filter sterilized solution of 250 mg Lysing enzymes from *Trichoderma harzianum* (Sigma) in 10 ml KCl buffer (0.6 M KCl and 50 mM CaCl_2_) containing 10% fetal bovine serum (FBS). The lysing solution was incubated for 2 h at 30°C, shaking at 100 rpm. The protoplasts were then filtered through four layers of Whatman lens cleaning tissue and washed with KCl buffer and STC buffer (10 mM Tris HCl, 50 mM CaCl_2_, 1.0 M sorbitol, pH 7.5). The protoplasts were collected by centrifugation at low speed, after which the supernatant was carefully removed. After washing, the protoplasts were dissolved in STC buffer at a concentration of 1–10 × 10^6^ protoplasts/ml. 100 μl of this solution was mixed with 2 μg transforming DNA (or 2× 1 μg for co-transformations), and 20 μl 50% PEG 6000 solution (in STC buffer). The mix was incubated for 30 min at 4°C after which 200 μl PEG was added and the mixture was incubated for another 10 min at 4°C. The transformation mixture was spread onto MH agar containing 1.0 M sorbitol and supplemented with 20 μg/ml hygromycin B (Sigma) as a selection marker. The transformation plates were incubated for 1–2 weeks at 37°C. For the first two days, the plates were incubated facing up, due to the large volume of the transformation mixture. This allowed enough time for the plate to absorb the transformation mixture. After two days the plates were turned upside down for the remaining incubation time.

### Characterization of the transformants

Successful integration of the hygromycin selection marker in Mm55 was confirmed via PCR with primer pair hygR_FW/hygR_RV (647 bp). Integration of the GFP reporter was confirmed with primer pair GFP_FW/GFP_RV (570 bp). To confirm the transformed strain was indeed *M*. *mycetomatis* and not a contaminant, a *M*. *mycetomatis* species-specific PCR was also performed with primer pair 26.1A/28.3A (420 bp) [10].

To screen the transformants for green GFP fluorescence, a small square of the edge of the colony was excised and inverted on a slide containing a droplet of medium. Images were taken with an Olympus-IX51 inverted fluorescence microscope, using a 100× objective with oil immersion. CellSens software (Olympus) was utilized to acquire images, which were then processed with the Fiji image processing package of ImageJ [11].

### Pathogenicity of the transformant in *Galleria mellonella*

Larvae of the greater wax moth *G*. *mellonella* were used to assess the pathogenicity of the GFP strain and to examine the composition of the grains. To prepare the inoculum, the fungus was grown on SAB agar for two weeks, then the mycelium was harvested, sonicated for 30 s at 10 μm and incubated at 37°C in RPMI (Roswell Park Memorial Institute) medium supplemented with 0.35 g/L L-glutamine, 1.98 mM morpholinepropanesulfonic acid (MOPS) and 100 mg/L chloramphenicol. After two weeks, the biomass was harvested, weighed, and suspended in phosphate buffered saline (PBS) to achieve a concentration of 8 mg/mL. The fungal suspension was sonicated for 2 min. at 10 μm. The larvae were infected with 4 mg of fungal suspension and survival was investigated daily over a period of 10 days. In addition, for the Mm55-GFP infected larvae, samples for histopathological evaluation were taken 4 h, 24 h, 72 h and 7 days post infection. The larvae were fixed in 10% buffered formalin for 48h, dissected longitudinally using a scalpel and fixed for another 48h before further routine histological processing [12]. The two halves were stained with either hematoxylin and eosin (H&E) or Grocott methenamine silver. To assess the fungal burden, grains were manually counted by two independent scientists and scored based on size as either small, medium, or large as described by Lim et al. [13]. The sum of all grains represents the total amount of grains, the total size of the grains represents the size of the grains multiplied by minimum size of their respected category [13].

### Statistical analysis

Larvae survival lines were compared using the Log-rank test with GraphPad Prism 8 (version 8.2.0, GraphPad Inc.). To determine the difference in grains and grain sizes between the larvae infected with the transformant and the control, a Mann-Whitney test was performed. A p-value smaller than 0.05 was deemed significant.

## Results

### *M*. *mycetomatis* is highly sensitive to hygromycin B

**Fig 2:**
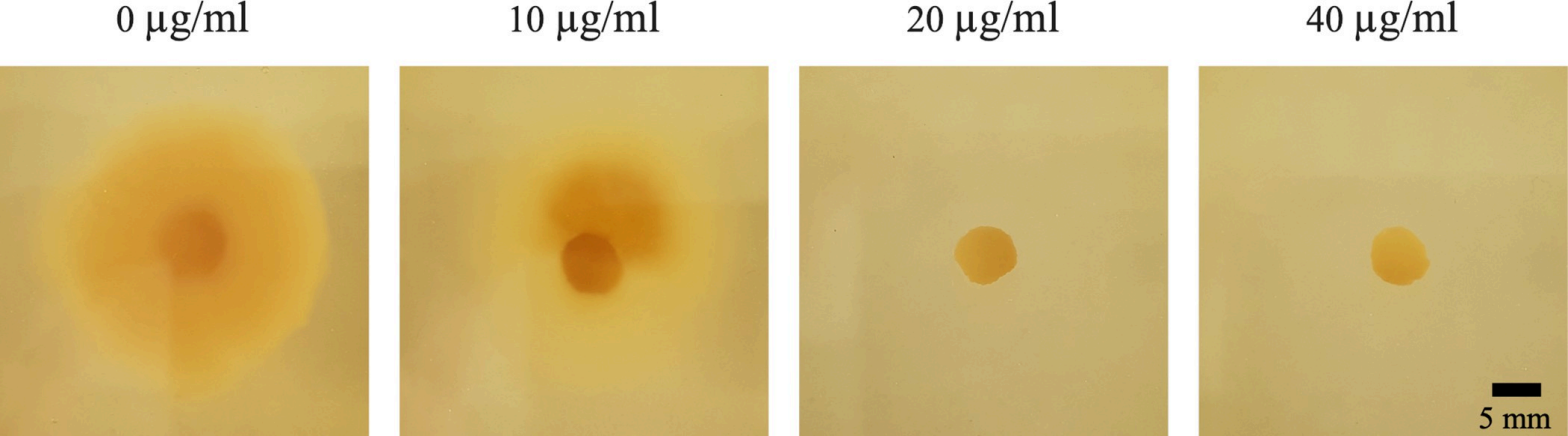
Hygromycin B growth assay with *M*. *mycetomatis* strain Mm55. Mm55 is highly susceptible, with no growth observed at a low concentration of 20 μg/ml.

In order to be able to develop a transformation technique, the first step was to identify a suitable selection marker. Hygromycin B is an antibiotic that is commonly used as a selection marker in a variety of fungal species [7]. *M*. *mycetomatis* strain Mm55 was highly sensitive to hygromycin B with reduced growth already observed at a concentration of 10 μg/ml compared to 0 μg/ml (Fig 2). No growth was observed from a concentration of 20 μg/ml hygromycin. These results indicate that hygromycin can be used as a selection marker for *M*. *mycetomatis*.

### The *M*. *mycetomatis gpd* promoter as a driver for hygromycin resistance

Plasmid pAN7-1 contains the hygromycin resistance gene (*hph*) from *E*. *coli* under control of the *Aspergillus nidulans gpdA* promoter and *trpC* terminator. Several transformation attempts of *M*. *mycetomatis* strain Mm55 with pAN7-1 did not lead to any hygromycin-resistant transformants. We hypothesized that the *A*. *nidulans gpdA* promoter driving *hph* was perhaps not working efficiently in *M*. *mycetomatis*. Therefore, we sought to optimize the expression of *hph* for *M*. *mycetomatis* by using a native promoter to drive its expression.

**Fig 3:**
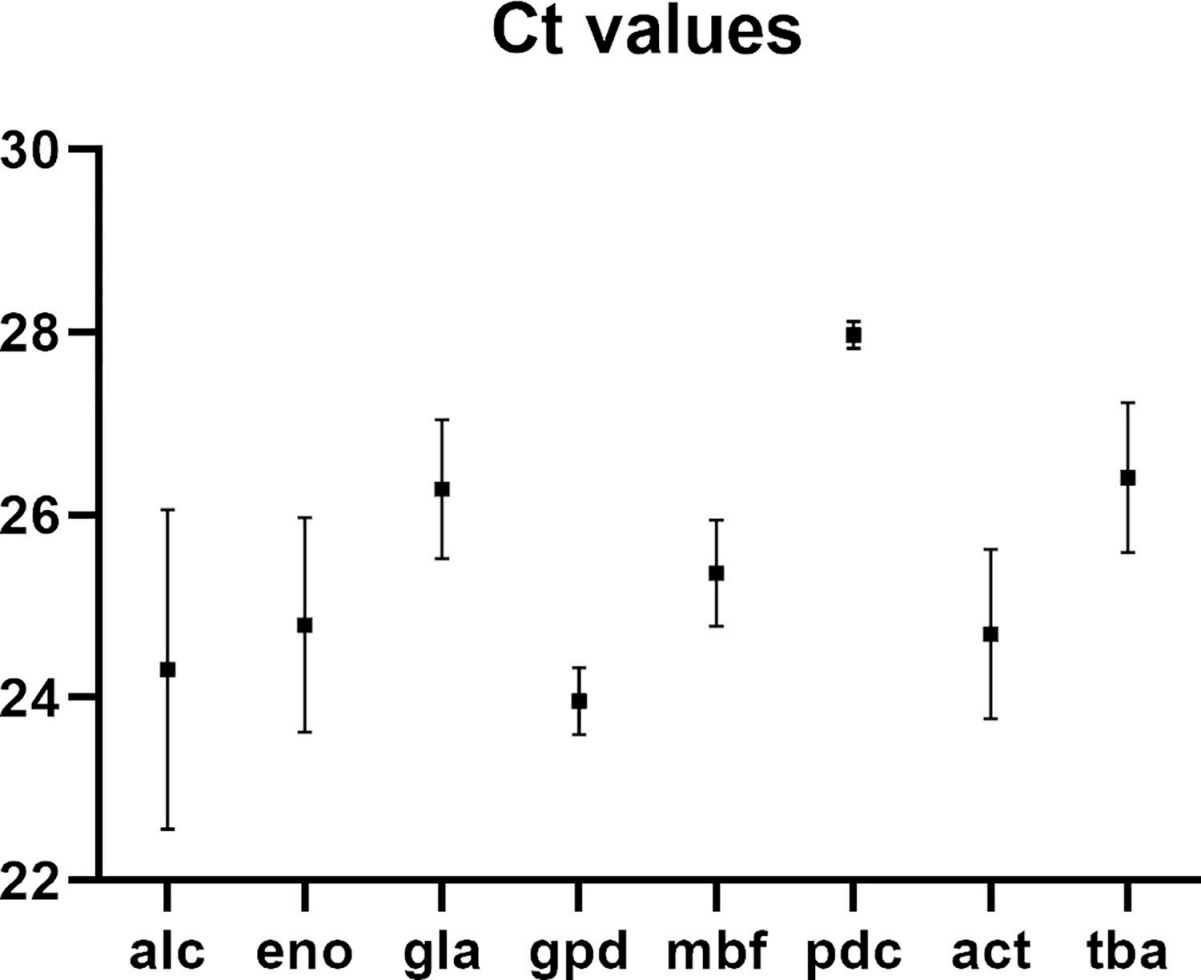
Ct values for the eight genes whose promoters were considered for expression of the selection marker gene. *Gpd* had the lowest mean Ct value. The raw data behind this figure are shown in S4 Table.

qPCR was used to determine which of the *M*. *mycetomatis* orthologues of constitutively expressed genes in other fungal species were highly expressed (S1 Table). Lowest Ct-values were observed for glyceraldehyde-3-phosphate dehydrogenase (*gpd*), followed by alcohol dehydrogenase (*alc*), actin (*act*), enolase (*eno*), multibridging factor (*mbf*), galactosidase (*gla*), tubulin (*tba*) and pyruvate decarboxylase (*pdc*) (Fig 3) (S4 Table).

Based on these results, the *M*. *mycetomatis gpdA* promoter was chosen to drive the expression of *hph*. The promoter in pAN7-1 was replaced with a 1.5 kb promoter region of the *M*. *mycetomatis* Mm55 *gpdA* gene. In addition, the terminator in pAN7-1 was replaced with a 0.5 kb terminator region of the native *M*. *mycetomatis* Mm55 *trpC* gene (KXX79020). The resulting plasmid in which *hph* is under control of a native *M*. *mycetomatis* promoter and terminator was named pSDP002.

### Hygromycin resistance as a selection marker for the genetic manipulation of *M*. *mycetomatis*

**Fig 4:**
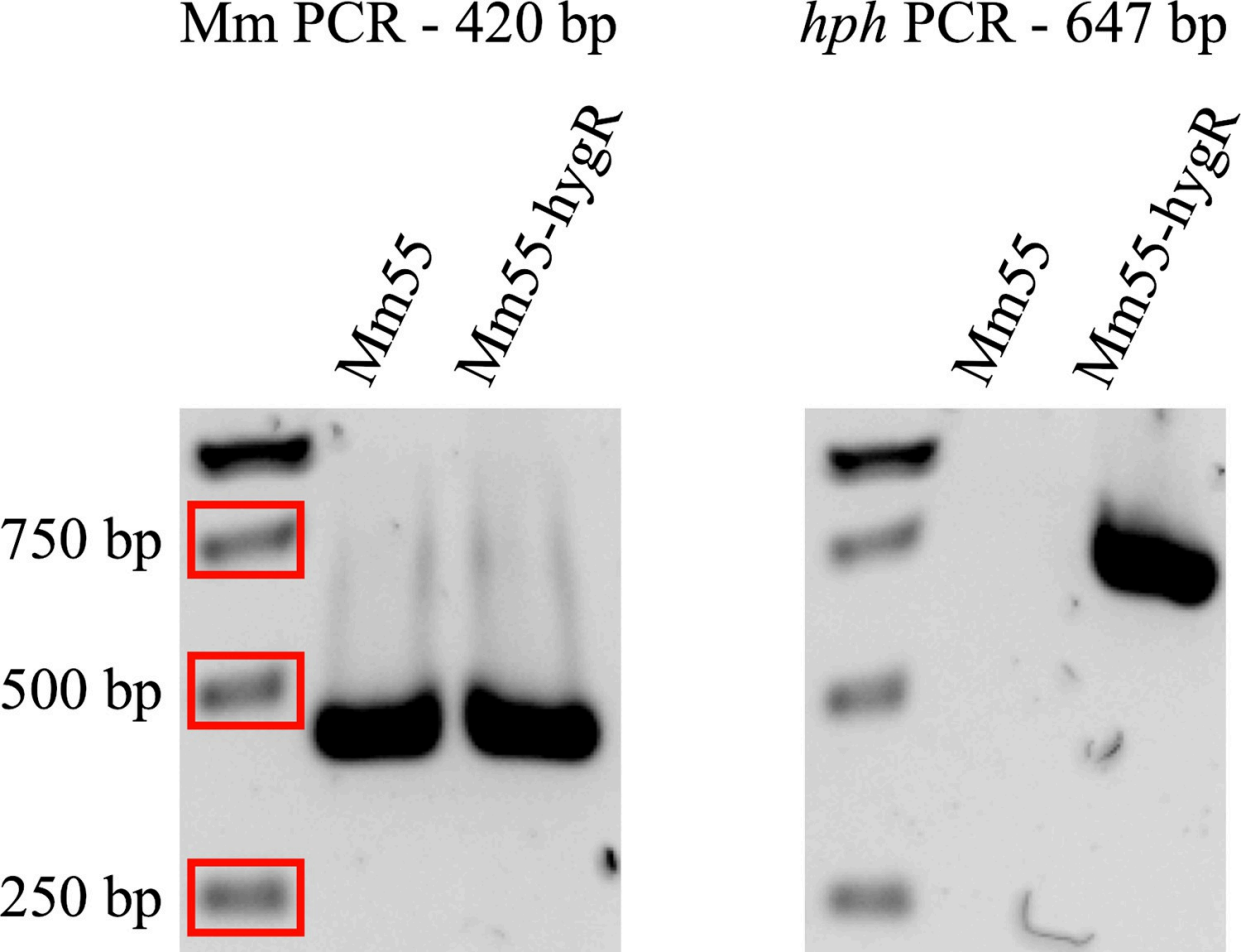
Characterization of Mm55-hygR via PCR. On the left an *M*. *mycetomatis* species-specific PCR to confirm that Mm55-hygR is not a hygromycin-resistant fungal contaminant. Both Mm55 and Mm55-hygR were positive, as expected. On the right a PCR against *hph* to confirm its integration. Only Mm55-hygR was positive, as expected, since wildtype Mm55 does not carry *hph*.

pSDP002 was transformed into Mm55 via a protoplast-mediated transformation method as described in the Material and Methods section. The transformation mixture was spread onto MH agar plates containing 20 μg/ml hygromycin. As a control, untransformed protoplasts were spread onto MH agar plates without hygromycin (positive control) and MH agar plates with hygromcyin (negative control). After two weeks, growth was visible on the positive control plate, but not on the negative control plate, indicating that untransformed protoplasts could not grow on hygromycin, as expected (see Fig 1). On the transformation plate, growth was visible, indicating that the transformation was successful. This was validated by PCR analysis of a *M*. *mycetomatis* species-specific gene and *hph* (Fig 4). The species-specific PCR was positive for both wildtype Mm55 and hygromycin resistant transformant Mm55-hygR, excluding the possibility that Mm55-hygR was a hygromycin-resistant contaminant. The *hph* PCR was negative for wildtype Mm55 and positive for Mm55-hygR, confirming that the transformation led to successful integration of *hph* into the genome of Mm55-hygR. The transformation was repeated six times and positive transformants were obtained each time. On average 3 transformants were obtained per 1 × 10^5^ protoplasts (see S5 Table). In summary, the *hph* construct optimized for expression in *M*. *mycetomatis* can be used successfully as a selection marker for the transformation of *M*. *mycetomatis*.

### GFP as a reporter for *M*. *mycetomatis*

**Fig 5:**
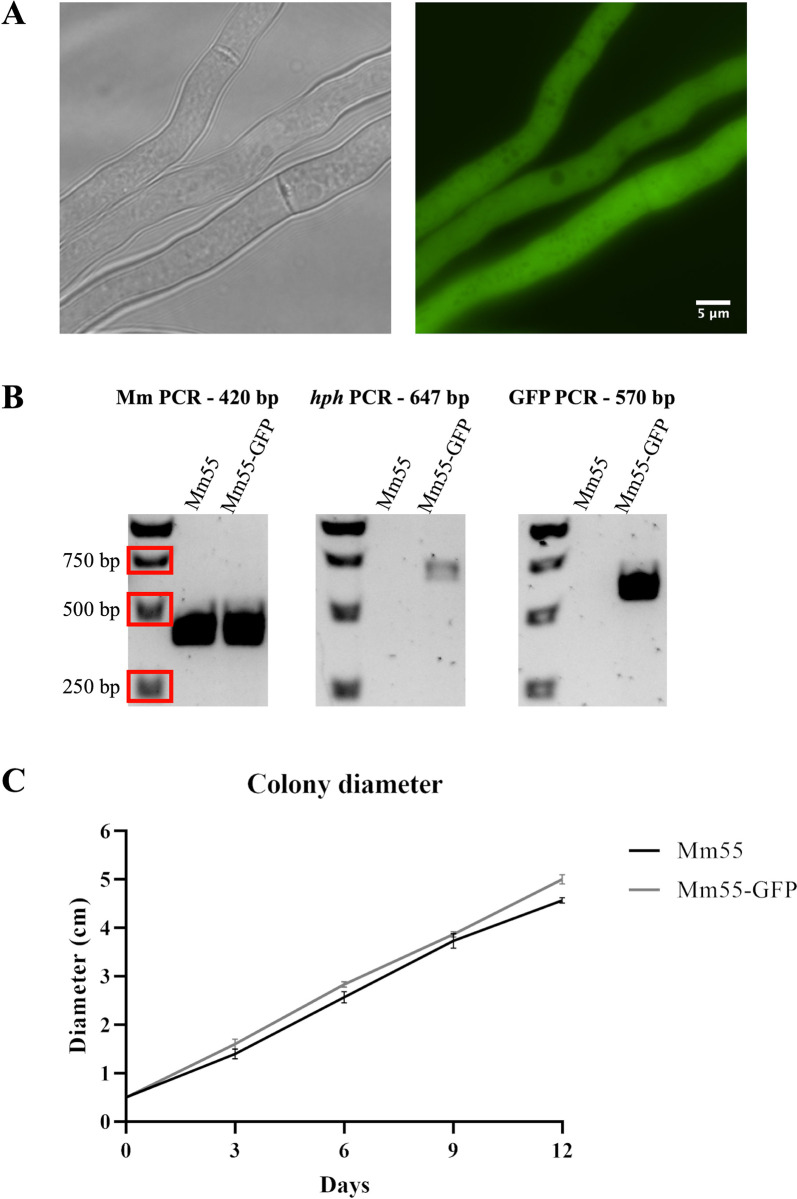
Characterization of *M*. *mycetomatis* cytoplasmic GFP-expressing strains. A) After co-transformation with pSDP002 and pDS1, one of the transformants showed fluorescence. B) The *M*. *mycetomatis* species-specific PCR confirmed the transformant was not a hygromycin-resistant contaminant and PCRs against the *hph* and *gfp* genes confirmed their integration into the genome of the transformant. C) The Mm55-GFP strain was growing at a similar rate as the wild-type Mm55 strain on SAB agar. Error bars indicate standard deviation, n = 3. Raw data presented in S6 Table.

To confirm the above described method for the genetic manipulation of *M*. *mycetomatis*, another transformation was conducted to create a cytoplasmic GFP-labelled *M*. *mycetomatis* strain. Plasmid pGPD-eGFP harbors a *gfp* gene under control of the *A*. *nidulans gpdA* promoter and *trpC* terminator. This plasmid was used to create a new plasmid, pDS1, in which the *gfp* gene was placed under control of the *M*. *mycetomatis* native *gpdA* promoter. A co-transformation with pSDP002 and pDS1 was conducted, as described in the Materials and Methods section, and after one week three colonies were visible on the transformation plate. The transformants were screened under a widefield microscope for green fluorescence and two out of the three colonies showed fluorescence (Mm55-GFP) (Fig 5A). After multiple rounds of cultivating, one of the colonies lost its fluorescence. The other colony still showing fluorescence was analysed by PCR, which confirmed the species and integration of both the *hph* and the *gfp* gene (Fig 5B). To assess the health of the Mm55-GFP strain, the colony diameter on agar plates was measured over 12 days, using Mm55 wildtype as a control (Fig 5C and S6 Table). The Mm55-GFP strain showed similar growth as the control, indicating that the strain is healthy and does not show any defects in growth. In summary, the described transformation protocol can be successfully applied for co-transformations and *gfp* can be expressed in *M*. *mycetomatis*, indicating it could be a suitable reporter for *M*. *mycetomatis*.

### Pathogenicity of the GFP expressing strain

**Fig 6:**
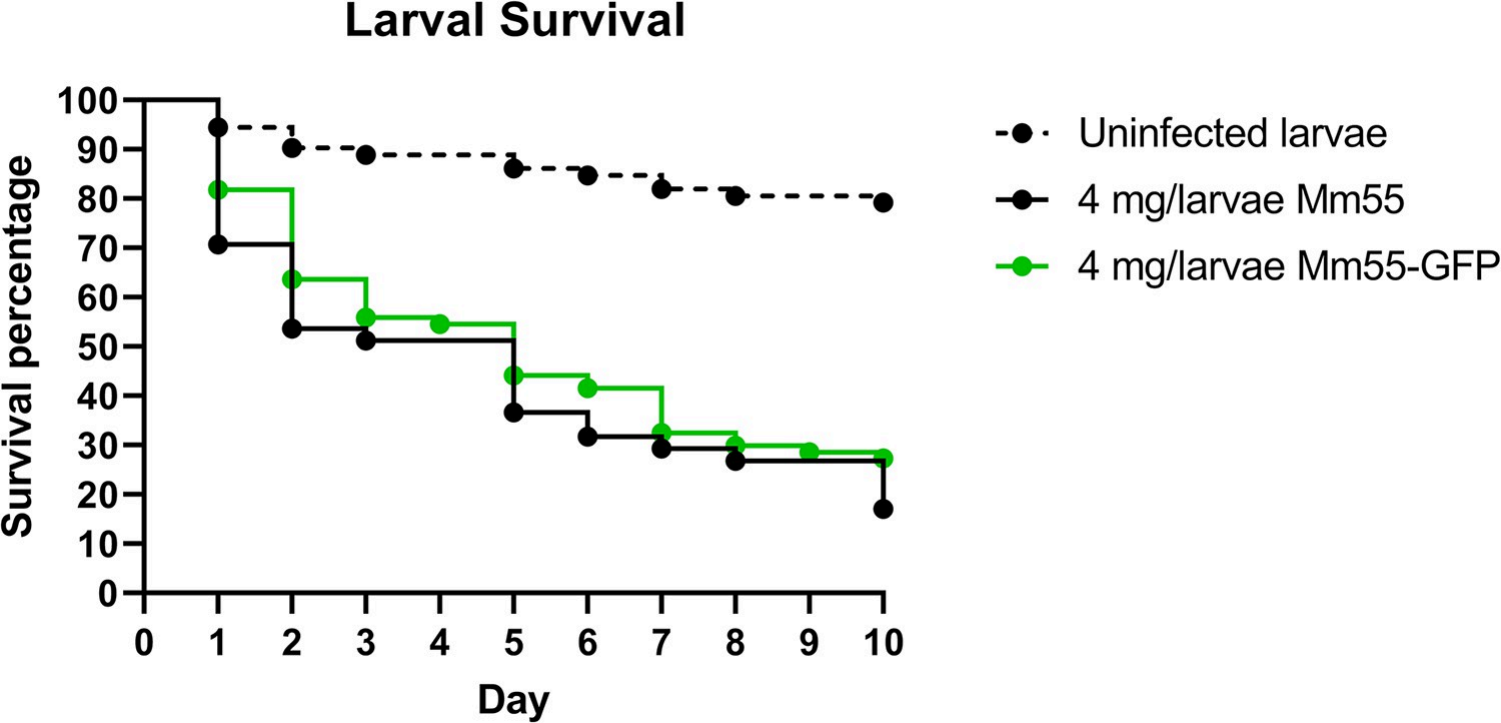
Survival of Mm55-GFP infected larvae and the burden of infection. A) Survival curve of Mm55-GFP (indicated in green, n = 77) and Mm55 wild type (indicated in black, n = 41) infected larvae. The dotted line indicates the uninfected PBS control (n = 72). The raw data is presented in Tabel S7. Both Mm55-GFP and Mm55 wild type significantly differ compared with the control (p = <0.0001 and p = <0.0001 respectively).

**Fig 7:**
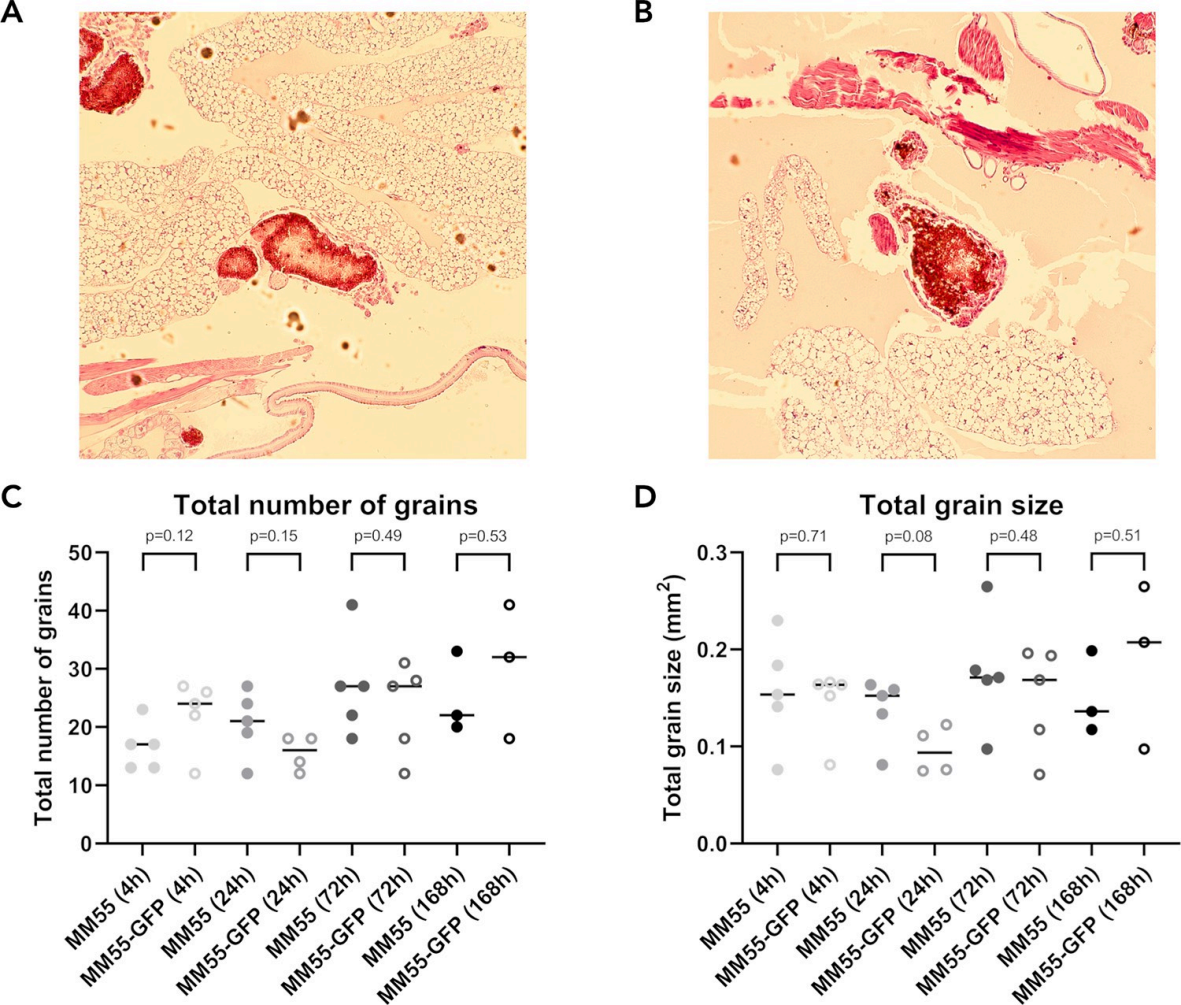
Grain comparison between Mm55 and Mm55-GFP. A) H&E stain of a grain in Mm55 wild type infected *G*. *mellonella*, 100 × magnification. B) H&E stain of a grain in Mm55-GFP infected *G*. *mellonella*, 100 × magnification. C&D) Burden of infection measured in the total number of grains counted (S8C Table) and the respective calculated total grain size (S9D Table). For each time point and strain n = 5, except for time point t = 24h for Mm55-GFP (n = 4) and t = 168h (n = 3) for both strains. p-Values are calculated per condition and time-point with Mann-Whitney. Values are shown above each time-point. No significant differences were noted in the total number of grains (C) or the total grain size (D).

To assess the pathogenicity of the GFP-labelled *M*. *mycetomatis* strain, *G*. *mellonella* larvae were infected with Mm55-GFP, using Mm55 wild type as a control. Here, no significant difference in larval survival was found, indicating that the Mm55-GFP strain did not lose its pathogenicity (p = 0.24) (Fig 6 and S7 Table). Furthermore, no visual differences in histopathological examinations were noted between Mm55-GFP and the wild type (Fig 7A and 7B and S8 and S9 Tables), indicating that Mm55-GFP was still able to form the characteristic mycetoma grains. The course of infection was further evaluated by scoring the burden of infection by assessing total number of grains and total grain size. The burden of infection for larvae infected with Mm55-GFP was compared with those of Mm55 wild type-infected larvae. No significant difference was found between Mm55-GFP and the wild type in the total number of grains, nor in total grain size, in any of the respective time points (Fig 7C and 7D).

## Discussion

A protoplast-mediated transformation method was applied to *M*. *mycetomatis*, the main causative agent of mycetoma. This method is based on a standard transformation protocol for *Aspergillus* spp., using hygromycin resistance, optimized for expression in *M*. *mycetomatis* in this study, as a selection marker. Transformation with this selection marker led to successful integration of *hph* into the *M*. *mycetomatis* genome. In addition, a co-transformation of the hygromycin selection marker with a GFP reporter construct, optimized for expression in *M*. *mycetomatis*, resulted in a cytoplasmic *gfp*-expressing *M*. *mycetomatis* strain, Mm55-GFP, which showed no significant decrease in pathogenicity. This reporter strain could in the future be used as a reporter strain for (microscopic) growth analyses, infection assays with animal models or cell cultures, and quantification of MICs. A GFP strain was already available for *Nocardia brasiliensis*, one of the causative agents of bacterial mycetoma (actinomycetoma) [14,15], meaning that GFP-expressing strains are now available for both eumycetoma (mycetoma caused by fungi) and actinomycetoma.

We observed a correlation between the protoplast concentration and the number of obtained transformants, with a higher starting amount of protoplasts leading to more transformants (S5 Table). To generate more protoplasts, more biomass is required, however, the difficulty with *M*. *mycetomatis* is the slow growth rate of this fungal species. A long period of growth is required to acquire a sufficient amount of biomass, however, as the mycelium ages the cell wall becomes thicker, making it more difficult to generate protoplasts. In the described protocol we cultured for no more than 7 days at each culturing step, to avoid the mycelium becoming too old. To obtain more biomass, a larger starting culture will be required to generate more protoplasts. In combination with an increased amount of transformation DNA, we hypothesize that this will lead to an increased transformation rate of the described transformation protocol.

Here we have described a transformation method for random integration of plasmids and the next step will be to create knock-out strains. In this case it will be useful to create an *M*. *mycetomatis* strain deficient for either the KU70 or the KU80 protein. These proteins are involved in homologous recombination and knocking either out has resulted in increased homologous recombination, i.e., increased number of positive transformants in other filamentous fungi [16,17]. We identified orthologues for both KU70 and KU80 in the genome of *M*. *mycetomatis*. In addition, it will be useful to generate a library of selection markers suitable for *M*. *mycetomatis*, including the generation of auxotrophic markers, using for example the *pyrG* gene coding for orotidine-5’-decarboxylase which is essential for *de novo* pyrimidine biosynthesis. Classic gene editing tools were used in this manuscript, but in the future we would like to implement the CRISPR-Cas9 genome editing technique to improve the generation of *M*. *mycetomatis* genetic clones [7].

Having a genetic toolbox available for *M*. *mycetomatis* will allow for detailed studies of virulence factors for *M*. *mycetomatis*. Multi-omics approaches have identified genes that are potentially important for the pathogenicity of this fungal species. For example, a zincophore was identified to be upregulated during the formation of grains [3]. This was recently further explored in a bioinformatic review, which revealed a number of interesting genes which could be analysed with this new tool [18]. Knock-out strains of such identified genes can be tested for grain formation in the *G*. *mellonella* infection model for mycetoma [19]. Besides the genes that play a role in pathogenicity, the genome of *M*. *mycetomatis* also contains interesting genes from a biotechnology perspective, such as a recently identified novel type of laccase [20]. A genetic toolbox will also aid the characterization of these genes as well.

In conclusion, a genetic modification method is described for *M*. *mycetomatis* for the first time. In addition, it was demonstrated that GFP can be used as a reporter, opening up possibilities to tag proteins and allow characterization of their behavior. Having these tools available will allow for more studies on the molecular mechanisms behind mycetoma infections. This knowledge will help improve therapeutic options to treat this debilitating disease.

### Data statement

All data is available in the manuscript and its supplementary files.

## Supporting information

S1 Table Primers for qPCR constitutively expressed genes. In this table the Genbank Accession numbers and the forward and reverse primer for each of the selected genes is listed.
(XLSX)

S2 Table Primers used for cloning and gene integration. In this table the forward and reverse primers used for cloning and gene integration are listed.
(XLSX)

S3 Table Plasmids used in this study. In this table a description and reference for each of the plasmids used in this study are listed.
(XLSX)

S4 Table Ct-values. In this table the Ct-values obtained for the eight genes whose promoters were considered for expression of the selection marker gene are shown. The data presented in this table was used to generate Fig 3 in the manuscript.
(XLSX)

S5 Table Transformation rate. In this the data from 6 transformation experiments is shown. For each transformation experiment, the number of protoplasts present in the transformation mix, the number of resulting transformants and the number of transformants/1,00E+05 protoplasts is shown. Based on these results the average transformation rate was determined.
(XLSX)

S6 Table Colony diameter. In this table, the colony diameter of 3 growth experiments performed with strain mm55 and strain mm55-GFP are shown. The data is used to generate Fig 5C. The diameter (cm) of each of the colonies is measured on day 0, day 3, day 6, day 9 and day 12.
(XLSX)

S7 Table Survival rate. The recorded survival of uninfected larvae, larvae infected with *M*. *mycetomatis* strain mm55 and larvae infected with *M*. *mycetomatis* strain mm55-GFP at day 0, day 1, day 2, day 3, day 4, day 5, day 6, day 7, day 8, day 9 and day 10. This data was used to generate Fig 6.
(XLSX)

S8 Table Grain count. In this table, the number of grains counted in histological sections of larvae infected with *M*. *mycetomatis* strain mm55 and larvae infected with *M*. *mycetomatis* strain mm55-GFP are shown. Grains were counted at time point, 4h, 24h, 72h and 168h after infection. This data was used to generate Fig 7C.
(XLSX)

S9 Table Grain size. In this table, the size in mm^2^ of grains counted in histological sections of larvae infected with *M*. *mycetomatis* strain mm55 and larvae infected with *M*. *mycetomatis* strain mm55-GFP are shown. The size was determined at time point, 4h, 24h, 72h and 168h after infection. This data was used to generate Fig 7D.
(XLSX)
